# Some Like It Hot: Camera Traps Unravel the Effects of Weather Conditions and Predator Presence on the Activity Levels of Two Lizards

**DOI:** 10.1371/journal.pone.0137428

**Published:** 2015-09-23

**Authors:** Chris Broeckhoven, Pieter le Fras Nortier Mouton

**Affiliations:** Department of Botany and Zoology, Stellenbosch University, Stellenbosch, South Africa; Universidad de la Republica, URUGUAY

## Abstract

It is generally assumed that favourable weather conditions determine the activity levels of lizards, because of their temperature-dependent behavioural performance. Inactivity, however, might have a selective advantage over activity, as it could increase survival by reducing exposure to predators. Consequently, the effects of weather conditions on the activity patterns of lizards should be strongly influenced by the presence of predators. Using remote camera traps, we test the hypothesis that predator presence and weather conditions interact to modulate daily activity levels in two sedentary cordylid lizards, *Karusasaurus polyzonus* and *Ouroborus cataphractus*. While both species are closely related and have a fully overlapping distribution, the former is a fast-moving lightly armoured lizard, whereas the latter is a slow-moving heavily armoured lizard. The significant interspecific difference in antipredator morphology and consequently differential vulnerability to aerial and terrestrial predators, allowed us to unravel the effects of predation risk and weather conditions on activity levels. Our results demonstrate that *K*. *polyzonus* is predominantly active during summer, when ambient temperatures are favourable enough to permit activity. In contrast, a peak in activity during spring was observed in *O*. *cataphractus*, with individuals being inactive during most of summer. While favourable weather conditions had a strong effect on the activity levels of *K*. *polyzonus*, no such relationship was present in *O*. *cataphractus*. Contrary to our hypothesis, the presence of terrestrial predators does not seem to affect daily activity levels or alter the influence of weather conditions on activity levels. We conclude that inactivity in *O*. *cataphractus* appears to be related to seasonal differences in vulnerability to predators, rather than the presence of predators, and highlight the importance of additional selective pressures, such as food abundance, in determining the species’ activity levels.

## Introduction

Activity provides individuals with the opportunity to feed, mate and defend territories and plays a vital role in determining survival and reproductive success [1–3]. In lizards, it has been hypothesised that the time of activity is synchronised with changes in weather conditions, especially ambient temperature [4–6], because the majority of physiological processes operate optimally near the preferred body temperature [7–9]. Field observations, however, suggest that additional selective pressures might influence daily activity levels [5,10,11]. Predation is presumably the strongest selective pressure to act against continuous activity, because it entails immediate fitness loss [12]. Hence, individuals often decrease activity when predators are active or when predation pressure is high [13,14]. As a result, the influence of weather variables on activity levels should be strictly regulated by predatory pressures and activity patterns should reflect the selective advantage of the positive and negative components of activity [5,14–16]. Few studies, however, have attempted to determine whether weather conditions and predator presence interact to produce variation in foraging behaviour and/or activity. For example, Lopez-Dariaz et al. [17] showed that *Anolis sagrei* did not forage near ground-level if the predatory lizard *Leiocephalus carinatus* was active, despite favourable weather conditions. Nevertheless, associations between long-term daily activity levels and weather conditions, and the interactive effect of predation risk, are seldom investigated.

The lack of studies on the activity patterns of lizards can be partially attributed to the difficulties associated with estimating activity levels. Traditionally, activity patterns have been inferred from direct observations of activity levels recorded for a short period of time [5,11,18]. In addition to the obvious limitations, the presence of an observer could alter the behaviour of organisms or predators thereof [19,20]. Recently, camera trapping technology has been used to calculate the activity budgets of mammals (reviewed in [21,22]) and has proven to reliably estimate activity levels [23]. Camera trapping is a non-invasive and less labour-intensive method to obtain activity data than focal observations. Consequently, activity patterns can be inferred from data collected from an extended continuous period of time, giving it a clear advantage over traditional observation methods. The use of camera traps in lizard studies, however, has been limited to monitor lizards (genus *Varanus)* [24,25] and their purpose restricted to obtaining estimates of site occupancy [24,25]. The restricted use of camera traps in lizard studies results from the fact that most lizards are relative small and therefore fail to trigger camera traps, leading to limited trapping success [26]. Moreover, lizards do not exhibit strong site fidelity, rendering continuous observation of individuals difficult to accomplish.

Two cordylid lizard species, namely *Ouroborus cataphractus* and *Karusasaurus polyzonus* provide an opportunity to test the effects of weather conditions and predation risk on long-term daily activity patterns using camera trapping techniques. Firstly, like most cordylid lizards, *O*. *cataphractus* and *K*. *polyzonus* are predominantly rock-dwelling sit-and-wait foragers [27–29] and usually take up an ambush position close to the shelter (e.g. rock crevice) from which they chase prey over short distances. Their sedentary nature thus allows for continuous photography of individuals, thereby circumventing some of the above-mentioned problems. Secondly, although the two species are closely-related [30] and have an overlapping distribution [31], they differ in several life-history traits relevant to our hypothesis. Most notable is the major interspecific variation in anti-predator morphology: *K*. *polyzonus* is a relatively fast, lightly armoured lizard, while *O*. *cataphractus* is a slow-moving heavily armoured lizard [32,33]. Heavy armour in *O*. *cataphractus* serves as protection against attacks from small terrestrial mammals [33] and most likely evolved to facilitate foraging excursions to termite nests away from the safety of the crevice [34,35]. Armour in cordylid lizards, however, is assumingly ineffective against the beaks and talons of aerial predators [34,36]. Instead, light armour which allows a fast retreat to the shelter is the preferred strategy against aerial predation [34,36]. Hence, the difference in anti-predator strategy and consequently vulnerability to the two types of predators may influence the activity levels of *O*. *cataphractus* and *K*. *polyzonus*.

In concordance with Lopez-Dariaz et al. [17], we hypothesise that predator presence and weather conditions should interact to modulate activity. We predict that the effect of weather conditions on lizard activity is different in the presence of predators than in the absence of predators. More specifically, we predict that, regardless of weather conditions, the presence of terrestrial predators will have a more negative effect on the activity levels of *K*. *polyzonus*, compared to *O*. *cataphractus*, while the presence of aerial predators will have a more negative effect on the activity levels of *O*. *cataphractus*, compared to *K*. *polyzonus*.

## Materials and Methods

### Study site

The study site, located 20 km north of Lambert’s Bay, Western Cape, South Africa was restricted to an isolated area of c. 0.02 km^2^ consisting of scattered sandstone outcrops. Leipoldtville Sand Fynbos vegetation, consisting of perennial grass (*Cladoraphis cyperoides*) and dwarf shrubs (e.g. *Galenia africana*, *Zygophyllum morgsana*) is present on the coastal plains [37]. This arid region falls within the winter rainfall zone of South Africa and is characterised by extensive vegetative ground cover (mainly annual Asteraceae) and a peak in arthropod abundance during spring (August to October), followed by a period of low arthropod availability and high ambient temperatures during summer (December to April) [38–39]. Annual rainfall is low (less than 200 mm), but the close proximity of the study site to the Atlantic Ocean (< 10 km) reduces the aridity greatly. The study site was privately owned and verbal permission was obtained from the landowners prior to the initiation of data collection.

### Estimation of lizard activity and predator presence

**Fig 1:**
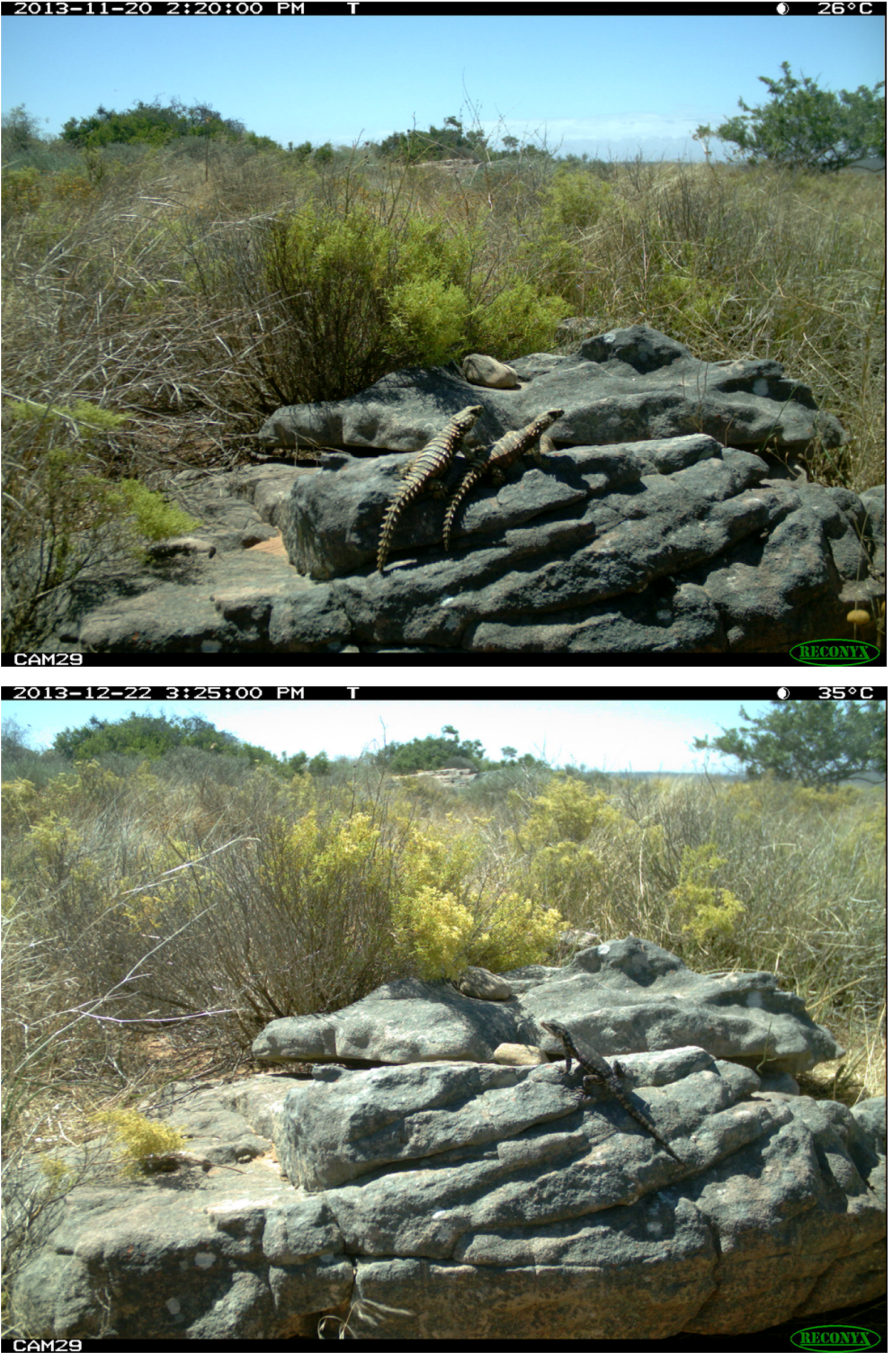
Example of a camera trap photograph used to calculate activity levels. Images illustrate active individuals of the heavily armoured *Ouroborus cataphractus* (top) and the lightly armoured *Karusasaurus polyzonus* (bottom).

Remote camera traps (Reconyx PC900 HyperFire, Reconyx Inc., Wisconsin, USA) were used to assess temporal variation in activity patterns. Cameras were mounted onto sand-colour painted metal poles, 80 cm above ground and positioned 1–2 m from a rocky outcrop inhabited by either one or both species. First, lizard activity was continuously recorded at ten rocky outcrops for four weeks, after which all cameras were positioned at different rocky outcrops. After three months (i.e. from 1 January 2013 till 31 March 2013), we presumed that the majority of the lizards and predators in the area were familiar with the camera traps. Following the familiarisation period, five rocky outcrops were selected based on the following criteria: (1) the rocky outcrop was required to be inhabited by both *O*. *cataphractus* and *K*. *polyzonus*, (2) the rocky outcrop was required to be a loose standing rock and (3) the rocky outcrop was required to have a simple crevice, with an opening on only one side of the rock. Furthermore, to account for the influence of group-size on activity in *O*. *cataphractus* [40], rocky outcrops were selected that were inhabited by different numbers of individuals. From 1 April 2013 till 31 March 2014, the cameras were programmed to take photographs every five minutes, from 07.00 h till 20.00 h. An example of a camera trap photograph used to calculate activity is provided in Fig 1. In addition, the five cameras deployed to record lizard activity levels were also programmed to detect the presence of predators. To ensure minimal disturbance, camera traps were checked for errors and data were offloaded only every 4–8 weeks.

### Calculation of lizard activity levels

Each day was divided into 24 consecutive half-an-hour intervals, with each interval being represented by six images. For each interval, we recorded the highest number of ‘active’ lizards. A lizard sitting with its body exposed outside the crevice, either mobile or immobile, was counted as ‘active’. In this sense, activity included a range of behaviours, including thermoregulation, feeding, mating and territory defence. The numbers of active individuals were summed up and divided by the total number of intervals (i.e. 24) to obtain a daily index of activity. In *O*. *cataphractus*, the total number of intervals was multiplied with the total group size in order to take the degree of sociality into account. This method allowed us to obtain an index of activity score ranging from 0 to 1, with 0 being no lizards active during the day and 1 being all lizards active during the entire day. Note that no discrimination was made between individuals within groups of *O*. *cataphractus* as we were interested in interspecific variation in activity patterns, rather than individual variation in activity.

### Meteorological data

Weather data were obtained from the Nortier weather station, 10 km south of the study site (S1 File). Weather variables included mean daily temperature, mean daily humidity, total daily rainfall, mean daily wind speed, mean daily barometric pressure and daily photoperiod.

### Data analyses

Time-series analyses were performed using Eviews version 8.1 (Quantitative Micro Software, Irvine, CA, USA) to determine the effect of weather variables, predator activity and their interaction effect on lizard activity. Prior to statistical analyses, we tested for stationarity of the data by examining each variable separately for the presence or absence of a unit root (i.e. indication of non-stationarity) using the Augmented Dickey-Fuller test. Non-stationary time-series indicate that the values increase or decrease over time. As such, they violated the assumptions of the statistical estimation theory and are unsuitable for regression analysis [41,42]. All variables, however, were stationary (S1 Table) and could therefore directly be used as input for time-series analysis.

The relationship between the average index of lizard activity (i.e. dependent variable), weather condition and predator absence/presence (i.e. independent variables) was investigated by performing ordinary least squares regressions. Because of the correlated nature of weather variables, prior to statistical analyses, a principal components analysis (PCA) on a correlation matrix with the raw (untransformed) weather variables and arcsine-transformed mean daily humidity was performed in the Statistical Package for the Social Sciences (SPSS) Statistics 17.0.1 (SPSS Inc., Chicago, IL, USA) and the resulting PC-scores were retained and used as input for the time-series analyses. Since weather, and potentially activity, tend not to change drastically from one day to the next, weather and activity variables from one day to the next might be highly correlated. Consequently, the Durbin-Watson statistic and Breusch-Godfrey Serial Correlation LM test was used to test for autocorrelation in the residuals from the regression analysis. The Durbin-Watson statistic ranges from 0 (positive autocorrelation) to 4 (negative autocorrelation), with a value of 2 indicating that no autocorrelation is present in the sample [43]. If the Durbin-Watson statistic was below or above 2, the autocorrelation function (ACF) and partial autocorrelation function (PACF) of the residual series were examined and the correct model was included in the regression analysis. A Breusch-Godfrey Serial Correlation LM test was then conducted to confirm the absence of serial correlation in the final model. The regression coefficients and significance levels were used to determine the strength of the effects of weather conditions and predator presence/absence on lizard activity.

## Results

### General lizard activity patterns

Despite the occurrence of missing data due to battery failure or damage to camera traps caused by livestock, lizard activity levels were recorded during 280 days (Table 1) on average per rock (S2 File). The camera trapping yielded a total of 223 860 photographs that served as input for the analyses of activity patterns. Due to the size of the rock, we were not able to reliably estimate the daily activity levels of the third *K*. *polyzonus* individual and this individual was excluded from subsequent analyses.

**Fig 2:**
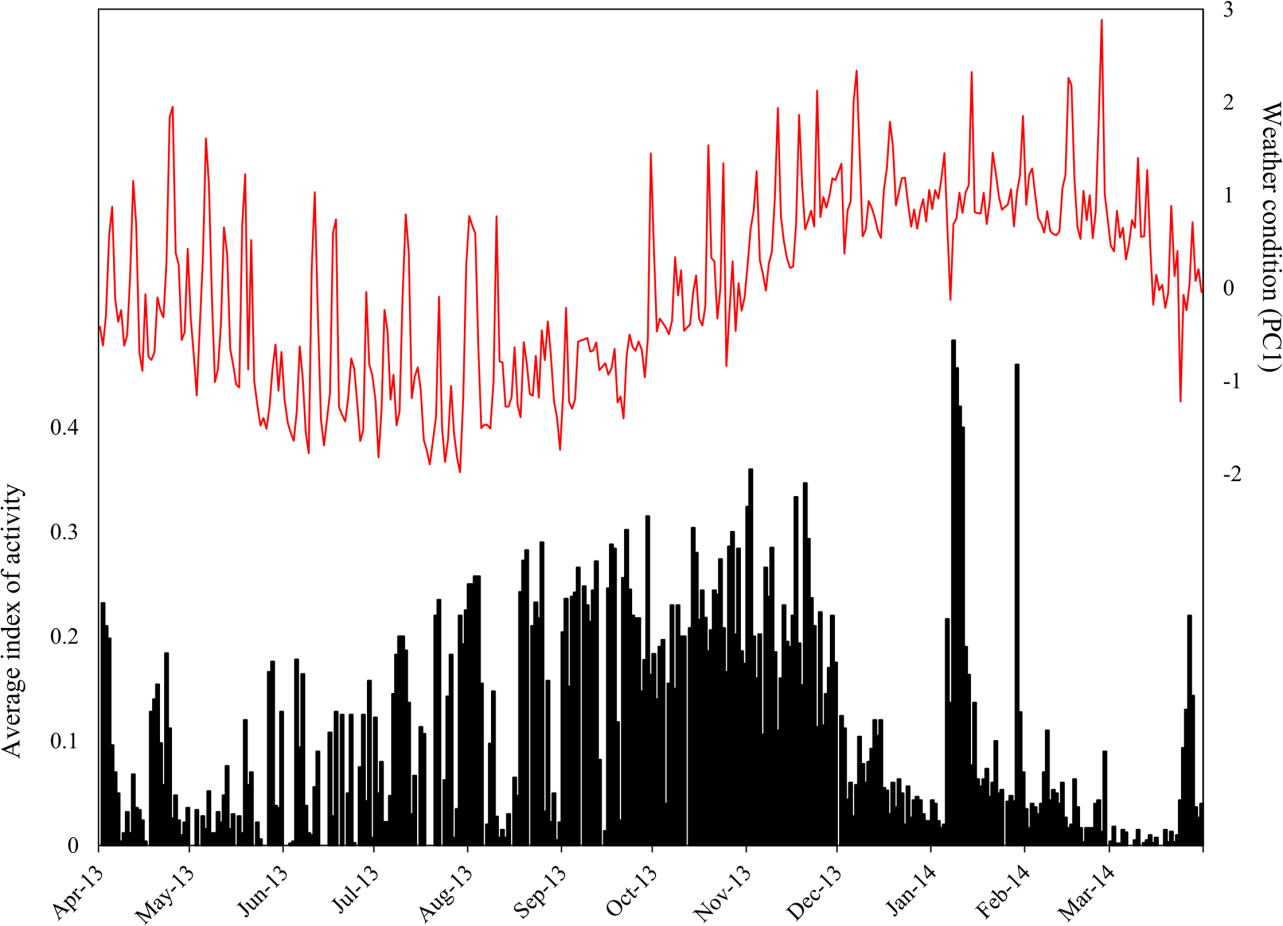
Activity pattern of *Ouroborus cataphractus*. A graph of the average index of activity plotted against weather condition (represented by PC1) illustrates that the activity levels of *O*. *cataphractus* are not determined by favourable weather conditions.

**Fig 3:**
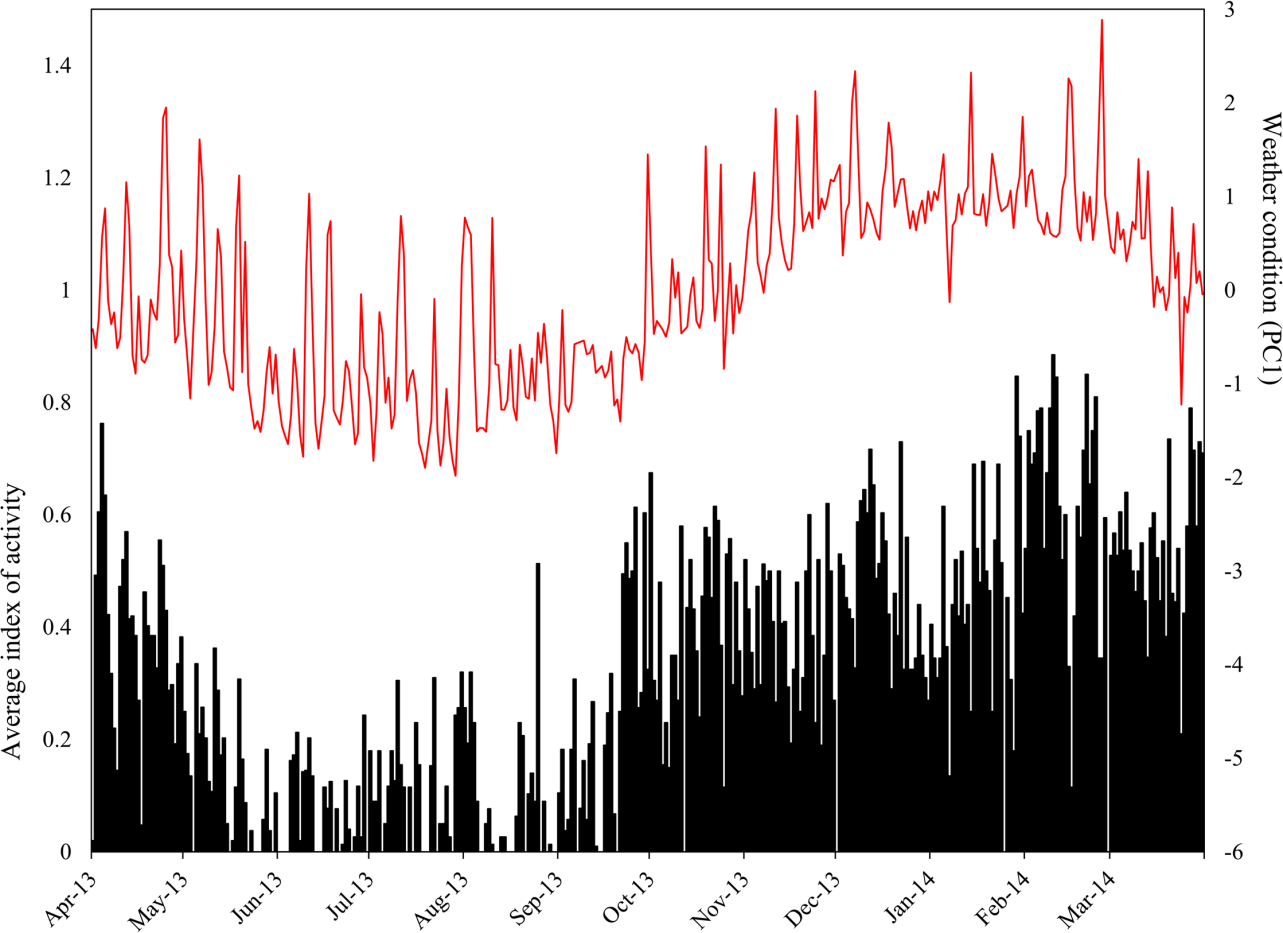
Activity pattern of *Karusasaurus polyzonus*. A graph of the average index of activity plotted against weather condition (represented by PC1) illustrates that high activity levels of *K*. *polyzonus* coincide with favourable weather conditions (high PC1 scores).

Individuals of *O*. *cataphractus* were predominantly active from late-August till the beginning of November, coinciding with spring season (Fig 2). During winter, days of inactivity and activity alternate each other, while during summer, lizards remained inactive most of the time. However, during January and March, several peaks of high activity were detected (Fig 2), coinciding with occasional summer rainfall. In *K*. *polyzonus*, an opposite pattern was present: activity was low to absent from late autumn until spring, but increased during late spring and peaked during summer months (Fig 3).

### Predator activity

**Fig 4:**
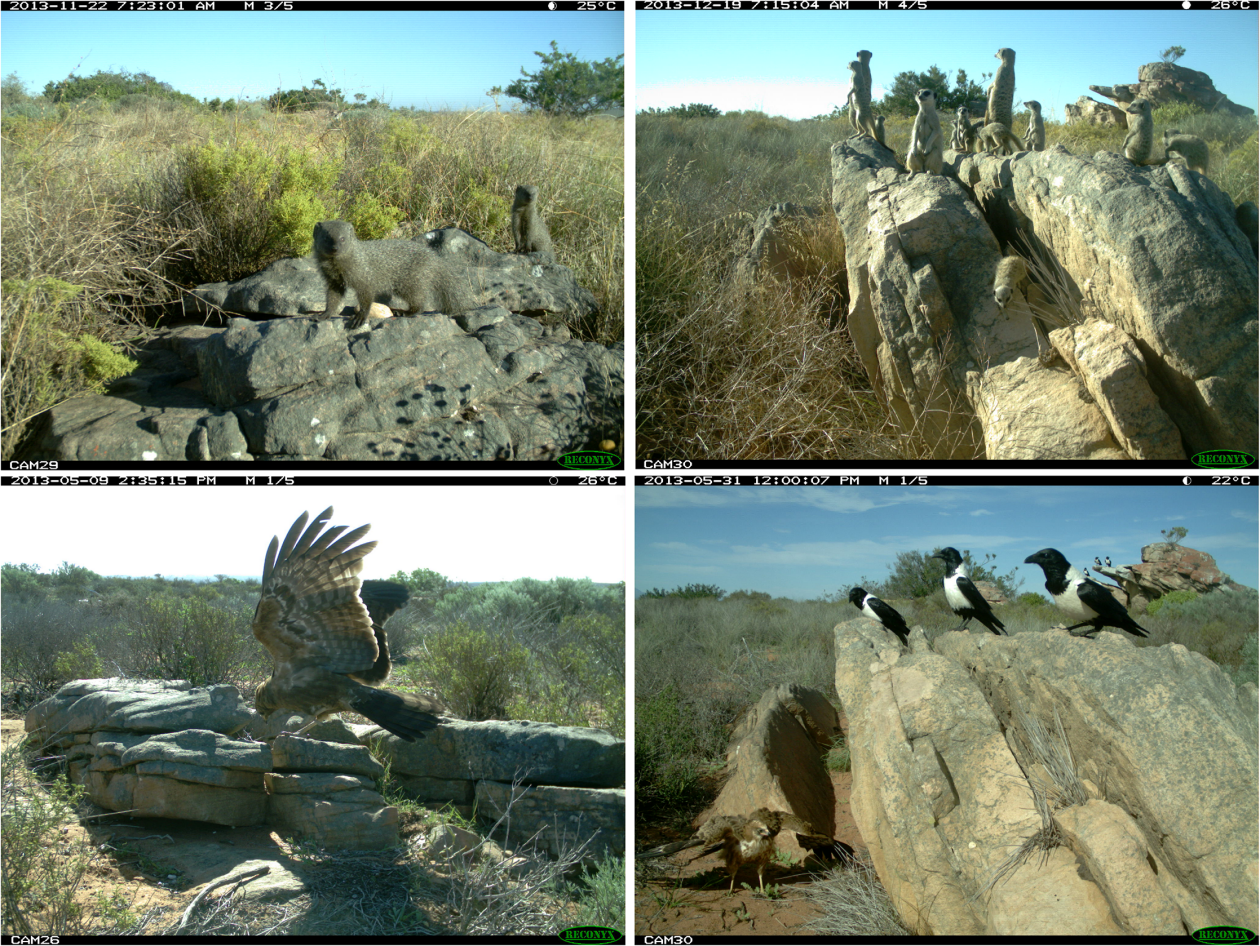
Camera trap photographs illustrating examples of terrestrial and aerial predators present in the habitat. Top: small grey mongoose (*Galerella pulverulenta*) and meerkat (*Suricata suricatta*), bottom: African harrier-hawk (*Polyboroides typus*) and pied crow (*Corvus albus*).

Remote camera trapping showed the presence of several terrestrial and aerial predators that can be classified as potential predators of the two species (Fig 4). Terrestrial predators were observed foraging close to lizard crevices on 122 days (Table 1), while aerial predators were detected by the camera traps on 11 days (Table 1). Small grey mongoose (*Galerella pulverulenta*) and meerkat (*Suricata suricatta*) were the most frequently observed terrestrial predators, while the African harrier-hawk (*Polyboroides typus*) and pied crow (*Corvus albus*) were the aerial predators that visited the lizard rocks.

**Table 1:** Frequency of visits by terrestrial and aerial predators to lizard rocks.

	Total days recorded	Terrestrial predators	Aerial predators
**Rock 1**	264	11	2
**Rock 2**	236	13	3
**Rock 3**	349	25	6
**Rock 4**	252	8	2
**Rock 5**	334	90	4
**Total**	356	133	11

The number of days on which terrestrial and aerial predators were detected, is indicated.

### Effects of weather conditions and predator presence on activity levels

Due to the low frequency of aerial predator occurrence (Table 1), all time-series analyses were limited to terrestrial predators. The ordinary least squares analysis showed that favourable weather conditions (represented by PC1; Table 2, S2 Table) had a strong positive effect on the activity levels of *K*. *polyzonus*, but not on the on the activity levels of *O*. *cataphractus* (Table 3, S2 Table). The presence or absence of terrestrial predators did not affect the influence of weather conditions on activity (all interaction effects: *P* > 0.05; Table 3, S2 Table). A significant effect of predator presence and interaction effect was present in *K*. *polyzonus* individual 2 (Table 3, S2 Table), but this is most likely due to the low number of camera trapping days (Table 1).

**Table 2:** Summary of the results from a principal component analysis performed on the weather variables.

	PC1	PC2
**Temperature**	**0.917**	0.009
**Humidity**	**-0.590**	**0.683**
**Barometric pressure**	**-0.668**	**-0.629**
**Wind speed**	0.337	**-0.623**
**Photoperiod**	**0.736**	0.423
**Rainfall**	-0.327	0.386
**Total variation explained**	39.98	26.30
**Eigenvalue**	2.398	1.578

Two principal components, explaining 66.3% of the total variation, were retained from the PCA analysis. The first axis was used to represent favourable weather conditions. This axis was positively correlated with temperature and photoperiod and negatively with humidity and barometric pressure. This axis represents a gradient from hot dry days with a long period of sunshine typically observed during summer to cold, humid days with less sunshine characterising winter. The negative correlation with barometric pressure represents thermal lows present in arid environments during the summer. The factor coordinate correlations and eigenvalues of the variables are shown. Values in bold represent loading scores greater than 0.50.

**Table 3:** Summary of the results from an ordinal least squares analysis on the activity time-series.

	*Ouroborus cataphractus*			*Karusasaurus polyzonus*		
	*β*	*t*	*P*	*β*	*t*	*P*
**Rock 1**						
** Predator presence**	-0.009	-0.424	0.67	0.089	1.648	0.10
** PC1**	0.015	1.978	0.05	0.059	3.027	**0.003**
Pp*PC1	0.035	0.553	0.58	0.053	0.819	0.41
**Rock 2**						
** Predator presence**	0.014	1.603	0.11	0.191	3.665	**0.0003**
** PC1**	0.006	1.142	0.25	0.077	5.101	**<0.001**
Pp*PC1	0.013	0.896	0.37	0.141	2.453	**0.015**
**Rock 3**						
** Predator presence**	0.010	0.347	0.73	-	-	-
** PC1**	-0.005	-0.623	0.53	-	-	**-**
Pp*PC1	-0.039	-1.279	0.20	-	-	-
**Rock 4**						
** Predator presence**	0.030	1.851	0.07	0.002	0.106	0.92
** PC1**	0.021	1.541	0.12	0.054	3.391	**<0.001**
Pp*PC1	-0.011	-0.729	0.47	-0.0004	-0.002	0.99
**Rock 5**						
** Predator presence**	0.004	0.354	0.72	0.011	0.380	0.70
** PC1**	0.007	1.29	0.20	0.033	1.825	0.07
Pp*PC1	-0.015	-1.101	0.27	0.014	0.376	0.71
**Species Average**						
** Predator presence**	0.012	1.599	0.11	0.036	2.409	**0.02**
** PC1**	0.009	1.392	0.16	0.036	2.851	**0.005**
Pp*PC1	-0.008	-1.117	0.26	-0.002	-0.137	0.89

The relationship between each independent variable (i.e. weather condition (PC1) and predator presence) and the dependent variable (i.e. lizard activity), as well as the interaction effect between predator presence and weather condition, is shown. Statistically significant *P*-values are indicated in bold. Legend: *β*, regression coefficient; *t* = *t*-test statistic.

## Discussion

It is widely assumed that the activity levels of lizards are regulated by favourable weather conditions, especially ambient temperature. [4–6]. Recent evidence, however, suggests that predation might act against continuous activity [10,11] and alter the effects that favourable weather conditions have on activity levels [12,17]. Using remote camera trapping, we were able to acquire long-term data on the daily activity levels of the two cordylid lizards *Ouroborus cataphractus* and *Karusasaurus polyzonus* and test how weather variables affect activity patterns in the presence and absence of predators. In contrast to our hypothesis, the presence of predators does not seem to influence the daily activity levels of the two lizard species tested in this study. Moreover, while favourable weather conditions strongly affect the activity levels of *K*. *polyzonus*, this association is absent in *O*. *cataphractus*.

### Influence of predation on activity levels

Several selective pressures, including predation [5], have been proposed to induce inactivity in lizards. Although prey respond to the presence of a predator by fleeing into the nearest refuge [44], our results show that neither the daily activity levels, nor the influence of weather conditions on activity appear to be affected by the presence of predators. Yet, despite the high occurrence of terrestrial predators in the area, it must be noted that our camera trapping methodology might not have provided us with a representative idea of the presence of aerial predators. Hence, our results do not allow us to exclude a possible effect of aerial predation on activity levels. For example, during summer, a lack of vegetative cover might increase visibility to aerial predators and consequently the aerial predation risk perceived by individuals [45]. Assuming that speed is the best antipredator strategy against predatory birds [34,36], the heavily armoured *O*. *cataphractus* might experience a higher aerial predation risk during the dry season compared to the lightly armoured *K*. *polyzonus*. Indeed, the number of attacks on stationary models of *O*. *cataphractus* by aerial predators was significantly higher during the dry season than during spring (see S3 File). Consequently, the cost of activity outside the safety of the retreat site might be high in *O*. *cataphractus* during summer, due to its vulnerability to aerial predators, and it would be advantageous for individuals to remain inactive.

The prolonged period of inactivity during summer, however, seems to be compensated for by a peak in activity during late-winter and spring. The extensive vegetation cover provided by annual plants during spring [39], might reduce visibility to aerial predators and decrease the aerial predation risk ([45], S3 File). Moreover, the protective effect of heavy armour would diminish the terrestrial predation risk [33], thereby allowing individuals to exploit the abundance of arthropods away from the crevice during spring and build up energy reserves for summer [46]. In addition, the temporal overlap of mating [46] and intense foraging from mid-winter to spring appears to be vital for the survival of *O*. *cataphractus*, as it would restrict overall exposure to aerial predation to an absolute minimum [34,35]. On the contrary, high ambient temperatures during summer would allow for maximal running capacity in the lightly armoured *K*. *polyzonus* [7,9]. Fast running, in turn, would permit prey capture outside the safety of the retreat site during summer without a significant increase in aerial predation risk. The relatively low ambient temperatures during winter/spring and high preferred body temperature [47] might impair running capacity and render *K*. *polyzonus* vulnerable to terrestrial predation during this time of the year. Nevertheless, more data on foraging behaviour outside the retreat site, as well as the actual predation risk experienced by both lizard species are required to test the hypothesis that the interspecific variation in activity is related to differential vulnerability to predators.

### Additional selective pressures promoting inactivity

An alternative explanation for inactivity is the fluctuating food abundance in highly seasonal environments. Inactivity conserves energy, which might be beneficial when food abundance is low [5]. For instance, Ballinger & Ballinger [48] report that periods of severe food shortage underlie inactivity in *Sauromalus obesus* and *Scleroporus jarrovi*. Likewise, low food abundance during the dry season might explain the low levels of activity in *O*. *cataphractus*. Heavily armoured cordylid lizards appear to compensate for their reduction in running speed by remaining close to the rock shelter during general maintenance behaviour [32]. In addition, group-living behaviour evolved in *O*. *cataphractus*, presumably to provide individuals with an enhanced vigilance benefit and lower the impact of aerial predation at the retreat site [49]. Given that the species displays a sit-and-wait foraging strategy [28], intraspecific competition for similar food resources becomes a major cost for the group-living *O*. *cataphractus*, compared to a solitary *K*. *polyzonus* [34]. High intraspecific competition for food at the rock-crevice resulting from group-living behaviour and low food abundance during summer might thus be the main selective pressure driving inactivity in *O*. *cataphractus* [34,35]. In contrast and following the above-mentioned, favourable weather conditions would not only maximise running speed, but also prey capture speed [7–9], thereby allowing the lightly armoured *K*. *polyzonus* to chase and capture prey items, despite low abundance, without an increased risk of aerial predation.

In addition to the seasonal interspecific differences in activity levels, our results revealed two interesting patterns in *O*. *cataphractus*. Firstly, although lizards remained inactive during most of summer, the exceptionally high amount of rainfall in January resulted in several peaks in activity. Variation in precipitation could stimulate arthropod activity, including termites, especially in (semi-)arid environments [50–52]. The sudden increase in food availability might have had an overriding effect on the tendency to remain inactive, despite the increased aerial predation risk, to lower the intraspecific competition for food experienced during the dry season. Secondly, even though winter was characterized by low, unfavourable temperatures, activity was observed on warmer days in *O*. *cataphractus*. Truter [53] proposed that warm days during winter may promote termite activity and consequently, lizard activity and that suitable temperatures would aid in digestion of prey items [8]. It must be noted that *O*. *cataphractus* has the lowest preferred body temperature range of all cordylids evaluated to date [47]. Although thermoregulation may be compromised in group-living individuals as a result of competition for suitable basking places [34,53], it might be an adaptation to feeding at lower ambient temperatures. Given the thermal dependence of prey capture behaviour and digestive efficiency [8,54], a lower preferred body temperature would aid individuals in capturing and processing prey efficiently during winter and spring.

## Conclusions

In summary, our results show a strong relationship between favourable weather conditions and the activity levels of *K*. *polyzonus*, but no such relationship was present in *O*. *cataphractus*. Moreover, the presence or absence of a terrestrial predator does not seem to influence how weather variables affect the daily activity levels of the two species. Our results suggest that selective inactivity in *O*. *cataphractus* might be a consequence of increased intraspecific competition for food associated with group-living behaviour and increased vulnerability to aerial predation during summer.

## Supporting Information

S1 File DATASET. **Weather conditions experienced by lizards at the Lambert’s Bay field site from 1 April 2013 till 31 March 2014.** Values represent the photoperiod, daily average temperature, daily average humidity, daily average barometric pressure, daily average wind speed and total rainfall. The principal component scores resulting from a principal component analysis conducted on the weather variables are shown.
(XLSX)

S2 File DATASET. **Daily activity levels of *Ouroborus cataphractus* and *Karusasaurus polyzonus* at the Lambert’s Bay field site from 1 April 2013 till 31 March 2014.** The presence or absence of terrestrial and aerial predators is indicated.
(XLSX)

S3 File Estimation of predation pressure during spring and summer. (DOCX)

S1 Table Results of Augmented Dickey Fuller (ADF) unit root tests conducted on the activity time-series, principal component scores and predator presence/absence data. Stationarity of the data (i.e. no increase or decrease over time) occurs when the *t*-statistic value is below the critical value. Statistically significant *P*-values are indicated in bold.
(XLSX)

S2 Table Summary of tests for serial correlation. Durbin-Watson statistic and results from the Breusch-Godfrey Lagrange multiplier test for residual autocorrelation after inclusion of the model terms are presented. Legend: AR, autoregressive.
(XLSX)
