# Supplementary material for: Some Like It Hot: Camera Traps Unravel the Effects of Weather Conditions and Predator Presence on the Activity Levels of Two Lizards
Source: PLoS One. 2015 Sep 23;10(9):e0137428. doi: 10.1371/journal.pone.0137428 (PMC4580596; doi:10.1371/journal.pone.0137428)
Supplement: S3 File — (DOCX) [file pone.0137428.s003.docx]

**SUPPLEMENTARY MATERIAL FILE 3**

**Materials and Methods**

To test whether the attack rate by aerial predators on *Ouroborus cataphractus* individuals differed between seasons, we made use of replica models [1-4]. A two-fold latex mould was constructed from an adult preserved specimen of *O. cataphractus*. Two 75 mm steal cut nails were inserted into the mould to provide weight and steadiness. Next, polyurethane foam (Alcolin, Cape Town, South Africa) was sprayed into the mould and was allowed to dry in an incubator at 35°C for at least one hour. Models were removed from the mould, excess foam was cut away and models were spray-painted. In order to match the colouration of the models to the population they represent, spectrophotometric data were obtained from Truter [5] and models were painted accordingly. Models of *O. cataphractus* were deployed during spring (September 2012) and summer (March 2013). In total 200 models were placed: half of the models on a rock surface outside lizard shelters and half on a ground surface, with at least 2 meters between consecutive models (Figure S3.1). The bottom-side of each model was provided with a number and the GPS-coordinates of all models were taken so no visual objects that facilitate recovery (e.g. flags) had to be used. All models were recovered eight days post placement and checked for signs of attack.

To test for differences in predation pressure between the seasons, the frequency of attacks on replica lizard models was calculated and compared between dry and rain season, as well as between models placed on a rock surface and on a sand substrate. Therefore, contingency table analyses were used, conducted in R 3.1.1 (R Development Core Team, 2014).

**Results**

Replica models of *O. cataphractus* placed on sand substrate and rock surface were attacked at similar rates during each season (spring: Fisher Exact Test; *P* = 0.68, summer: *χ*² = 1.59, *P* = 0.21; Fig. S3.2). However, there was a seasonal effect on the proportion of attacked models depending on the substrate. Models placed on sand substrate were attacked more often during summer than during spring (*χ*² = 8.87, *P* = 0.003, Fig. S3.2), but the frequency of attacks on models was similar on a rock surface (*χ*² = 0.80, *P* = 0.37, Fig. S3.2).

**References**

1 Schneider CJ, Smith TB, Larison B, Moritz C. A test of alternative models of diversification in tropical rainforests: ecological gradients vs. rainforest refugia. PNAS 1999;96:13869-13873.

2 Diego-Rasilla FJ. Influence of predation pressure on the escape behaviour of *Podarcis muralis* lizards. Behav Process. 2003;63:1-7.

3 Vervust B, Grbac I, Van Damme R. Differences in morphology, performance and behaviour between recently diverged populations of *Podarcis sicula* mirror differences in predation pressure. Oikos 2007;116:1343-1352.

4 Daly BG, Dickman CR, Crowther MS. Causes of habitat divergence in two species of agamid lizards in arid central Australia. Ecology 2008;89:65-76.

5 Truter JC. Thermoregulation in a group-living lizard, *Cordylus cataphractus*: A temporal and spatial analysis. Unpublished M. Sc. Thesis, Stellenbosch University. 2011.

**Figures**


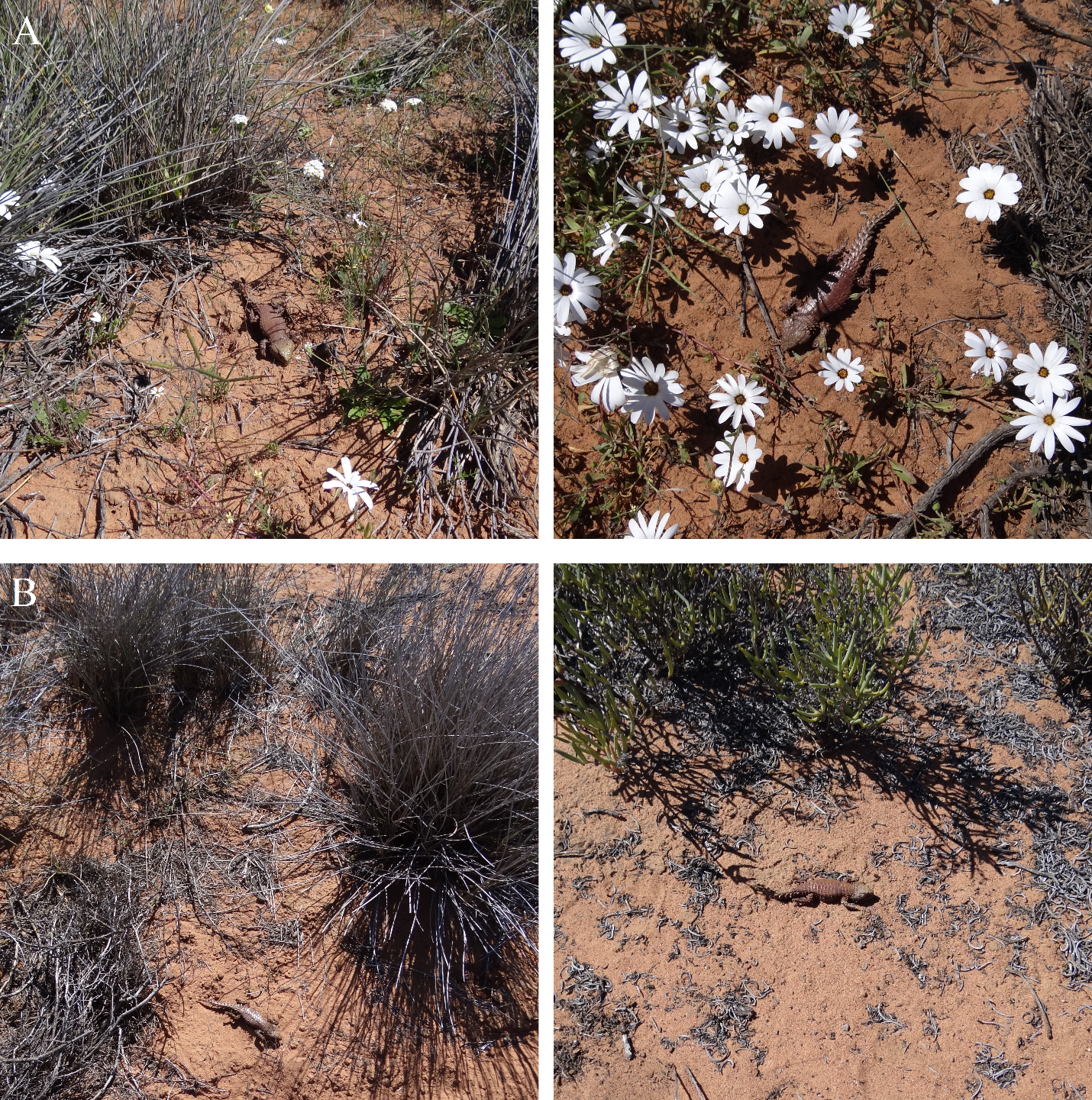


**Figure S3.1**: Photographs showing the placement of replica models of *Ouroborus cataphractus* on sand substrate to illustrate the differences in ground cover between spring (A) and summer (B).


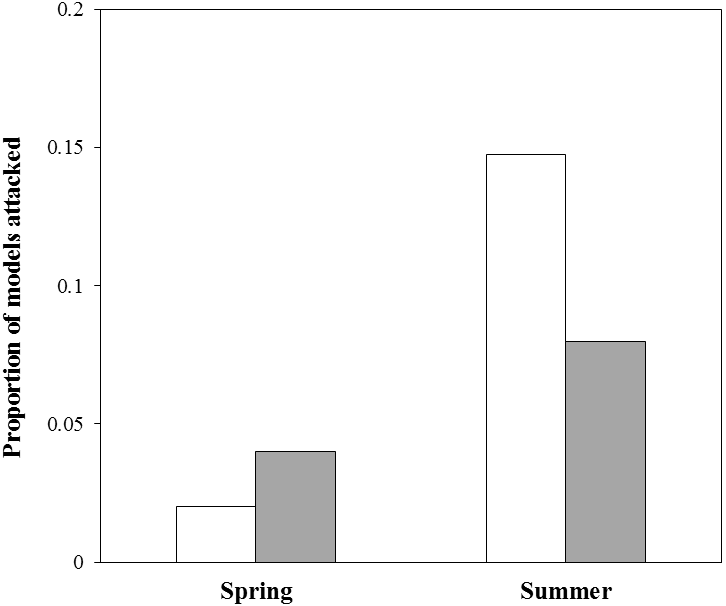


*

**Figure S3.2**: Differences in frequency of attacks on models placed on sand substrate in the open (□) and on a rock surface near crevices (■) during both seasons. The asterisk indicates a statistically significant difference at *P* < 0.05 in frequency of attacks on models.
